# DCK expression, a potential predictive biomarker in the adjuvant gemcitabine chemotherapy for biliary tract cancer after surgical resection: results from a phase II study

**DOI:** 10.18632/oncotarget.19037

**Published:** 2017-07-06

**Authors:** Sang Myung Woo, Kyong-Ah Yoon, Eun Kyung Hong, Weon Seo Park, Sung-Sik Han, Sang-Jae Park, Jungnam Joo, Eun Young Park, Ju Hee Lee, Yun-Hee Kim, Tae Hyun Kim, Woo Jin Lee

**Affiliations:** ^1^ Center for Liver Cancer, National Cancer Center, Goyang-Si Gyeonggi-Do, Korea; ^2^ College of Veterinary Medicine, Konkuk University, Seoul, Korea; ^3^ Department of Pathology, National Cancer Center, Goyang-Si Gyeonggi-Do, Korea; ^4^ Biometrics Research Branch, Research Institute, National Cancer Center, Goyang-Si Gyeonggi-Do, Korea; ^5^ Molecular Imaging Branch, Research Institute, National Cancer Center, Goyang-Si Gyeonggi-Do, Korea

**Keywords:** biliary tract cancer, gemcitabine, adjuvant therapy, chemotherapy

## Abstract

The role of adjuvant therapy following resection of biliary tract cancer (BTC) remains unclear. We therefore evaluated the feasibility and toxicity of adjuvant gemcitabine in patients with BTC. This clinical phase II trial was an open-label, single center, single-arm study. Within 8 weeks after gross complete resection of BTC, patients were started on intravenous infusions of gemcitabine 1000 mg/m^2^ over 30 min on days 1, 8, and 15 of every 28-day cycle. Intratumoral expression of cytidine deaminase (CDA), human equilibrative transporter-1 (hENT1), deoxycytidine kinase (dCK) and ribonucleotide reductase subunit 1 (RRM1) was measured by immunohistochemistry. This study enrolled 72 patients with BTC (26 with gallbladder cancer, 33 with extrahepatic cholangiocarcinoma, and 13 with intrahepatic cholangiocarcinoma). The 2-year recurrence-free survival (RFS) rate was 43% (95% CI, 33–57%). Multivariable analysis showed that DCK expression, vascular invasion, and lymph node metastasis were significantly associated with RFS. Twenty-one (31.8%) were positive for DCK immunoreactivity. The median RFS was 34.95 months for DCK-positive patients, compared with 11.41 months for DCK-negative patients. Although the primary hypothesis of this study, defined as a 2-year RFS of 60%, was not met, intratumoral DCK expression was significantly associated with RFS in patients with resected BTC treated with postoperative gemcitabine chemotherapy. Future randomized controlled trials are warranted.

## INTRODUCTION

Biliary tract cancer (BTC) has been defined as all tumors arising from the biliary tract or the biliary drainage system, including the intra- and extrahepatic bile ducts and the gallbladder. BTCs are generally rare and difficult to diagnose, and have an overall poor prognosis. Determination of optimal treatment regimens is therefore difficult. Although BTC is rare in Western countries [1], it is more common in Korea, accounting for 5.0% of all cancer deaths in 2012 [2]. Currently, surgery remains the only potentially curative treatment, although most patients develop tumor recurrence [3]. The 5-year survival rate after diagnosis is about 10% and has not increased over time [1, 4]. Despite extensive surgical resection, the 5-year survival rate of patients who have undergone curative resection is unsatisfactory, being 20–32% for intrahepatic cholangiocarcinoma, 30–42% for hilar cholangiocarcinoma, and 18–54% for distal cholangiocarcinoma [5]. Such poor outcomes provide a rationale for adjuvant strategies to improve survival. To date, few randomized controlled trials have evaluated the survival benefits of adjuvant therapy in patients with BTC. Rather, available evidence is mostly from retrospective case series and the results are often conflicting [6]. The guidelines of the National Comprehensive Cancer Network, based on a pooled analysis of 20 retrospective studies, recommend adjuvant treatment with 5-fluorouracil or gemcitabine, enrollment in a clinical trial, or best supportive care, depending on each patient's general condition [7]. This pooled analysis reported that adjuvant treatment had clinical benefit in patients with lymph node-positive and margin-positive disease [7].

Gemcitabine has generated particular interest as adjuvant therapy in patients with BTC because of its balanced benefit-toxicity ratio [8]. In addition, the combination of gemcitabine plus cisplatin represents the standard of care in patients with advanced disease [9]. To date, however, no prospective trials have investigated the role of adjuvant gemcitabine chemotherapy in patients with BTC.

Early identification of patients likely to be refractory to gemcitabine-based chemotherapy would be useful in clinical practice, where almost all advanced BTCs are treated with first-line gemcitabine-based chemotherapy. Thus, there is a need to identify markers of survival and of gemcitabine refractoriness in patients with BTC. Inherent and acquired resistance of cancer cells to gemcitabine may be determined by the levels of expression of genes involved in gemcitabine transport and metabolism. Several clinical studies have reported that intratumoral levels of human equilibrative nucleoside transporter 1 (hENT1), the major transporter responsible for gemcitabine uptake into cells, and of ribonucleotide reductase subunit 1 (RRM1) have predictive significance for survival in BTC patients treated with adjuvant gemcitabine therapy [10–13]. However, these were small-scale or retrospective studies. Our group recently reported that a polymorphism in the gene encoding cytidine deaminase (CDA) may predict the efficacy of gemcitabine-based chemotherapy in patients with advanced BTC [14]. Deoxycytidine kinase (dCK) is a key enzyme that activates gemcitabine by phosphorylation. Gemcitabine-resistant human cholangiocarcinoma cell lines showed downregulation of dCK [15]. In this study, we evaluated the efficacy and safety of adjuvant gemcitabine in patients with BTC. We also attempted to identify markers predictive of survival, by immunohistochemically analyzing the intratumoral expression of hENT1, dCK, RRM1, and CDA, to determine whether these levels were associated with the efficacy of gemcitabine against BTC. Furthermore, we analyzed eight single nucleotide polymorphisms (SNPs) of five genes to determine the relationships between these SNPs and clinical outcome in BTC patients treated with gemcitabine-based chemotherapy.

## RESULTS

### Patient characteristics

Between January 21, 2010, and July 28, 2014, 72 patients with BTC, including 26 with gallbladder cancer, 33 with extrahepatic cholangiocarcinoma, and 13 with intrahepatic cholangiocarcinoma, were enrolled (Table 1). Two patients with gallbladder cancer underwent a concomitant right hepatectomy, one underwent trisectionectomy, and all others underwent wedge resection of the gallbladder fossa. All 33 patients with extrahepatic cholangiocarcinoma underwent bile duct resection/reconstruction and supraduodenal lymphadenectomy; in addition, 17 patients underwent pancreaticoduodenectomy without liver resection and 15 underwent a major hepatectomy (right or left hepatectomy or extended hepatectomy). All surgical procedures for perihilar cholangiocarcinoma included caudate lobectomy. One patient with extrahepatic cholangiocarcinoma underwent a concomitant extended cholecystectomy. All 13 patients with intrahepatic cholangiocarcinoma underwent right or left hepatectomy, and one underwent a concomitant pancreaticoduodenectomy. All patients underwent dissection of the regional lymph nodes, but para-aortic lymph node dissection was not routinely performed. Proximal and distal ductal margins were assessed intraoperatively using frozen-tissue sections. If malignant cells were found in the ductal margin, the bile duct was further resected, to the maximum extent possible.

**Table 1:** Clinicopathological characteristics and univariate analysis of recurrence-free survival (RFS) in patients who received adjuvant gemcitabine chemotherapy after curative resection for BTC

Variables	*N* (event)	Univariable (RFS)	
		HR (95% CI)	*p* value
Age	72 (44)	1.01 (0.97–1.04)	0.656
Gender			
Female	24 (16)	1	
Male	48 (28)	0.79 (0.43–1.46)	0.455
Gross type			
Extrahepatic cholangiocarcinoma	33 (20)	1	0.253
GB cancer	26 (14)	0.98 (0.50–1.94)	0.957
Intrahepatic cholangiocarcinoma	13 (10)	1.81 (0.84–3.87)	0.128
Differentiation			
Well differentiated	18 (9)	1	0.166
Moderately differentiated	27 (17)	1.36 (0.61–3.05)	0.457
Poorly differentiated	20 (15)	2.24 (0.98–5.13)	0.057
Other	7 (3)	0.82 (0.22–3.03)	0.765
Tumor size (cm)	72 (44)	1.06 (0.91–1.23)	0.470
Vascular invasion			
Absent	47 (23)	1	
Present	25 (21)	3.27 (1.77–6.01)	< .001
Perineural invasion			
Absent	21 (8)	1	
Present	51 (36)	2.15 (1.00–4.66)	0.052
Lymphatic invasion			
Absent	25 (10)	1	
Present	47 (34)	2.81 (1.38–5.72)	0.004
T stage			
T1 + T2	43 (22)	1	
T3 + T4	29 (22)	2.07 (1.14–3.76)	0.017
N stage			
N0	40 (18)	1	
N1	32 (26)	3.35 (1.81–6.20)	< .001
ECOG performance status			
0	19 (12)	1	
1	53 (32)	0.71 (0.37–1.38)	0.312
Gemcitabine dosage	72 (44)	0.998 (0.996–1.000)	0.030
hENT1 (miss = 6)			
Negative	35 (26)	1	
Postive	31 (17)	0.71 (0.38–1.31)	0.267
dCK (miss = 6)			
Negative	45 (32)	1	
Postive	21 (11)	0.61 (0.31–1.22)	0.160
CDA (miss = 6)			
Negative	28 (17)	1	
Postive	38 (26)	1.43 (0.78–2.64)	0.251
RRM1 (miss = 6)			
Negative	24 (15)	1	
Postive	42 (28)	1.24 (0.66–2.32)	0.505

Tumors were identified as well differentiated, moderately differentiated, and poorly differentiated adenocarcinomas in 18 (25.0%), 27 (37.5%), and 20 (27.8%) patients, respectively. Vascular invasion, perineural invasion and lymphatic invasion were observed in 25 (34.7%), 51 (70.8%), and 47 (65.3%) patients, respectively. Thirty-two tumors (44.4%) were accompanied by lymph node metastases and 40 (55.6%) were not. All 72 patients underwent R0 resection.

### Efficacy and clinicopathologic variables

**Figure 1:**
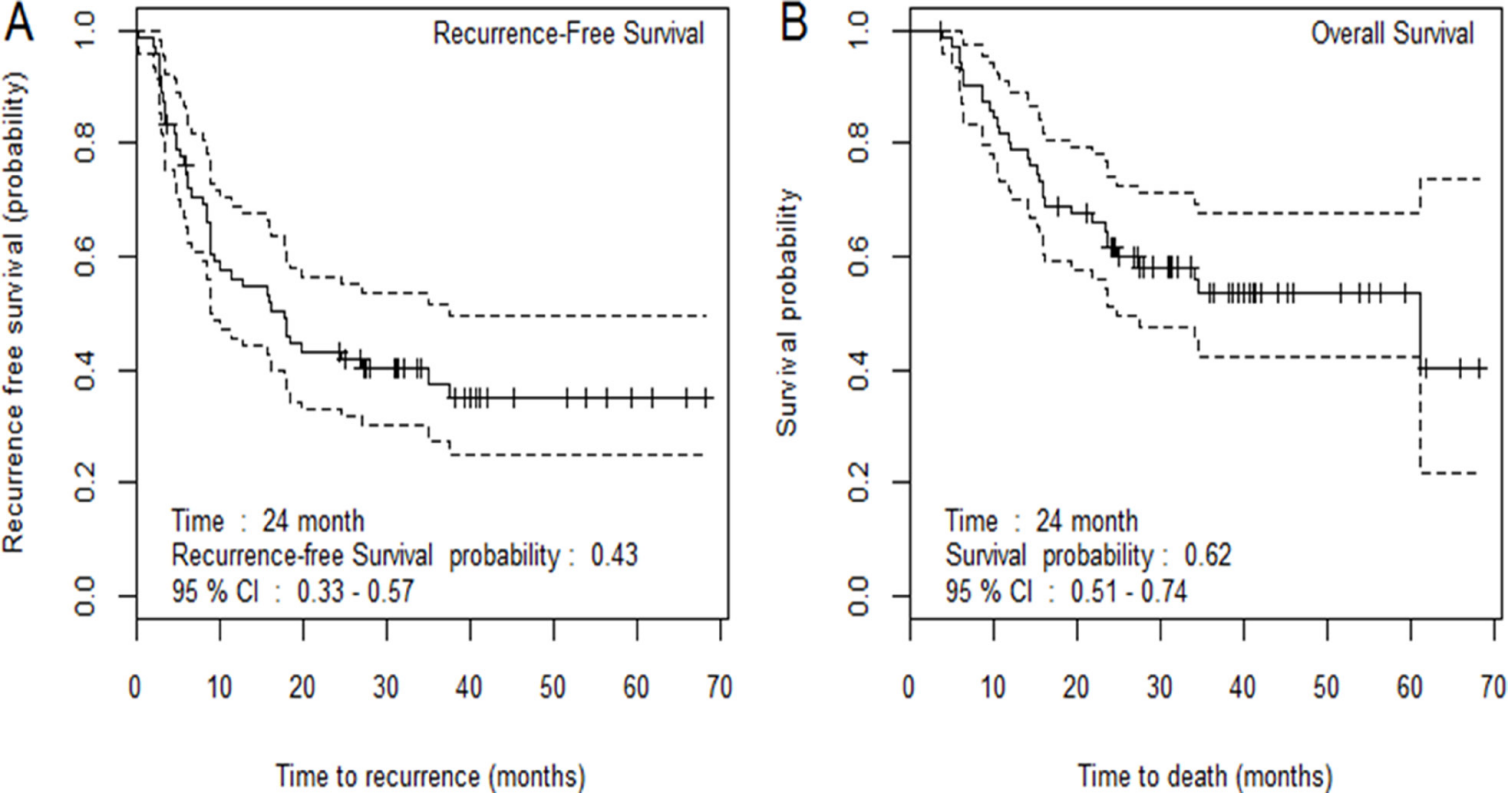
Recurrence-free survival (**A**) and overall survival (**B**) in all patients.

The median follow-up period for the entire study population was 38.07 months (range: 3.68–68.25 months), and the 2-year recurrence-free survival (RFS) rate was 43% (95% CI, 33% to 57%) (Figure 1). The median RFS was 17.59 months (95% CI, 9.17–37.55 months), and the median OS was 61.22 months (95% CI, 24.65 months–not yet reached). The 2-year RFS were 47% for extraheaptic cholangiocarcinoma, 49% for gallbladder cancer, and 23% for intrahepatic cholangiocarcinoma, respectively. However, the difference did not reach the statistical significance (Supplementary Figure 1). Univariable Cox proportional hazard analysis revealed that vascular invasion (*P* < 0.001), lymphatic invasion (*P* = 0.004), T stage (*P* = 0.017), gemcitabine dosage (*P* = 0.030) and lymph node metastasis (*P* < 0.001) were significantly associated with RFS (Table 1).

### Compliance and safety

Forty-seven (65.3%) of the patients completed all six cycles of planned adjuvant chemotherapy. Patients received a median of six cycles of chemotherapy (range, 1–6 cycles). The major cause of early termination was disease recurrence (13, 18%), followed by adverse events (*n* = 10, 14%), withdrawal of consent (*n* = 3, 4.1%), and Grade 5 toxicity (*n* = 1, 1.3%). There were no significant differences in distribution of adverse events between patients with and without tumor recurrence (Supplementary Table 1).

Of the 72 patients, 48 (66.7%) experienced Grade 3 or higher toxic events, including 41 (56.9%) patients who experienced hematologic toxicities and 15 (20.8%) who experienced non-hematologic toxicities. The most common hematologic toxicity was neutropenia, which occurred in 40 (55.6%) patients (Table 2). Grades 3/4 hematologic toxicities were more common than Grades 3/4 non-hematologic toxicities. The most common non-hematologic toxicities were nausea and increased total bilirubin level, which occurred in five patients (6.9%) each. There was one treatment-related death from pneumonitis.

**Table 2:** Frequency of adverse events

Toxicity Grade	0	1	2	3	4	5
Hematologic						
Anemia	1	38	29	4	0	0
Reduced platelet count	16	38	15	3	0	0
Reduced neutrophil count	7	0	25	32	8	0
Non-hematologic						
Nausea	25	33	9	5	0	0
Vomiting	47	16	7	2	0	0
Anorexia	34	29	7	2	0	0
Diarrhea	59	10	1	2	0	0
Febrile neutropenia	69	2	0	1	0	0
Abdominal pain	37	27	6	2	0	0
Asthenia	28	33	10	1	0	0
Elevated ALT	49	20	2	1	0	0
Elevated AST	44	24	3	1	0	0
T bilirubin	62	4	1	5	0	0
Pneumonitis	70	0	0	1	0	1
Edema	60	10	2	0	0	0
Myalgia	54	11	7	0	0	0
Dizziness	55	13	3	1	0	0
Headache	56	13	3	0	0	0

### Relationships between patient outcomes and intratumoral protein expression

**Figure 2:**
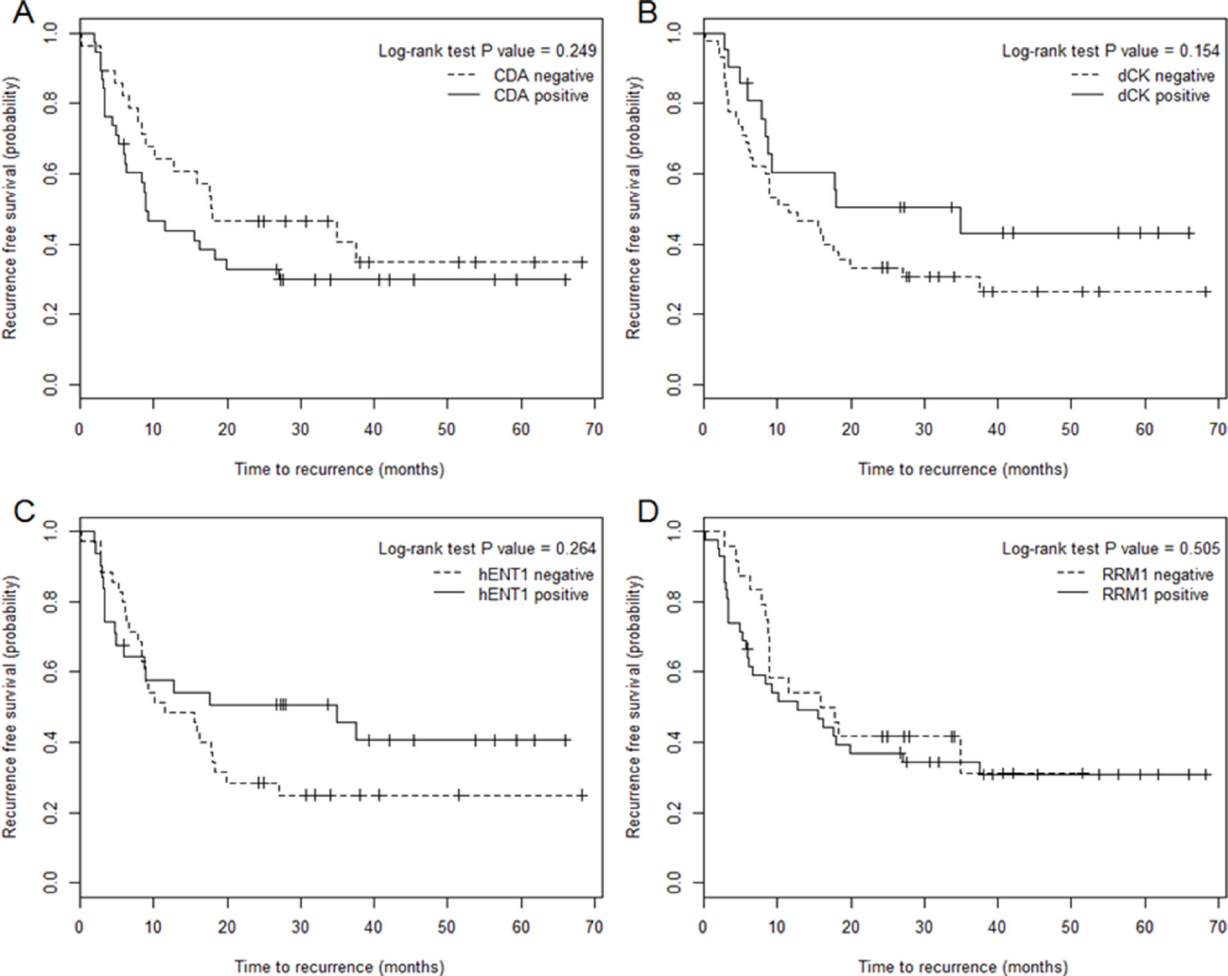
Relationships between recurrence-free survival and intratumoral protein expression CDA (**A**), dCK (**B**), hENT1 (**C**), and RRM1 (**D**) in biliary tract cancer.

To determine the effect of altered protein expression on gemcitabine sensitivity in BTC, we compared patients according to immunoreactivity for CDA, hENT1, dCK, and RRM1. Variables pre-selected based on univariable results (*P* < 0.2), including tumor differentiation, vascular invasion, perineural invasion, lymphatic invasion, T stage, N stage, and gemcitabine dosage, were entered to the multivariable model. In the multivariable model, DCK expression, vascular invasion, and lymph node metastasis were significantly associated with RFS (Table 3). Of the 66 samples analyzed, 21 (31.8%) were positive for DCK immunoreactivity. The median RFS was 34.95 months for DCK-positive patients, compared with 11.41 months for DCK-negative patients (Figure 2). However, the difference did not reach the statistical significance in the log-rank test (*P* = 0.154).

**Table 3:** Adjusted hazard ratio (95% confidence interval) and p-value of hENT1, dCK, CDA and RRM1 on recurrence-free survival (RFS)

			RFS					
Variables	hENT1		dCK		CDA		RRM1	
	HR (95% CI)	*p* value	HR (95% CI)	*p* value	HR (95% CI)	*p* value	HR (95% CI)	*p* value
Biomarker								
Negative	1		1		1		1	
Positive	0.62 (0.33–1.16)	0.131	0.49 (0.24–0.98)	0.043	1.09 (0.57–2.06)	0.795	0.96 (0.50–1.83)	0.895
Vascular Invasion								
Absent	1		1		1		1	
Present	2.29 (1.2–4.34)	0.012	2.39 (1.25–4.58)	0.009	2.19 (1.13–4.24)	0.020	2.24 (1.17–4.28)	0.015
N stage								
N0	1		1		1		1	
N1	2.92 (1.52–5.64)	0.001	2.96 (1.52–5.78)	0.001	2.68 (1.38–5.20)	0.004	2.74 (1.41–5.32)	0.003

* Variables to adjust were selected based on the univariable *p*-value < 0.2. Then these variables were included in the multivariable model together with each biomarker, and backward variable selection with an elimination criterion of *p*-value > 0.05 was applied. As a result, vascular invasion and N stage were adjusted for the evaluation of the adjusted hazard ratio of all four markers.

Fifty of the DNA samples were successfully genotyped. No discrepancies were observed between duplicate samples (–10%), and all genotyping data were included in the final analysis. None of the tested SNPs was significantly associated with RFS, or with Grade 3 or higher hematologic or non-hematologic toxicities (Supplementary Tables 2 and 3).

## DISCUSSION

The primary endpoint of this study, defined as an expected 2-year RFS rate of 60%, was not met. However, we found that intratumoral DCK expression was significantly associated with the RFS of patients with resectable BTC treated with postoperative gemcitabine chemotherapy. The clinical outcomes of patients in this study who received adjuvant gemcitabine chemotherapy were similar with those reported in a recent phase II trial of adjuvant capecitabine plus gemcitabine, followed by radiotherapy and concurrent capecitabine [16]. However, the efficacy of adjuvant gemcitabine can vary widely among individuals. Most published studies have been retrospective in design, resulting in potential biases. Nevertheless, the benefits of adjuvant therapy were found to be greatest in patients with factors associated with poor prognosis, including involved surgical margins or local lymph nodes. However, it remains unclear whether only higher risk patients benefit from adjuvant therapy or whether patient selection for treatment should be based on these factors [6]. Moreover, there are no validated biomarkers to date that can identify patients likely to benefit most from treatment [17].

Although the recommended standard adjuvant chemotherapy for advanced BTC consists of a combination of gemcitabine plus cisplatin [9], there have been few prospective trials. The therapeutic index should be considered in the adjuvant setting. Several previous clinical trials have suggested that patients with BTC who have undergone surgical resection with major hepatectomy are unable to tolerate the standard dose of gemcitabine [18, 19]. The risk-benefit ratio of adjuvant therapy should be optimized. Although it may be appropriate to extrapolate clinical trial findings to the adjuvant setting, differences in tumor burden along with physiologic differences after surgical resection of the primary tumor are biological confounders and important caveats in making such cross-comparisons.

Early identification of patient factors likely to enhance the efficacy and safety of gemcitabine-based chemotherapy would be useful in clinical practice, as almost all patients with advanced BTC are treated with first-line gemcitabine-based chemotherapy. Thus, there is a need to identify predictive markers in these patients. Previous retrospective studies reported that intratumoral hENT1 and RRM1 expression was significantly associated with outcomes in BTC patients who received adjuvant gemcitabine after surgical resection [10–13]. In the present study, however, only DCK expression displayed a significant relationship with disease response. Validation of these predictive biomarkers may enable gemcitabine chemotherapy to be tailored to individual patients, a key issue in developing effective treatment strategies of BTC.

DCK is the rate limiting enzyme for activation of deoxyribonucleoside prodrugs, which interfere with DNA synthesis and repair. The pharmacological activity of these nucleoside analogs is dependent on phosphorylation by DCK [20]. The expression of the dCK gene and protein and single-nucleotide SNPs in the *dCK* gene have been closely associated with gemcitabine chemosensitivity in patients with pancreatic cancer [21–23]. By employing immunohistochemistry, a small retrospective study found that high levels of dCK protein expression correlated positively with OS in pancreatic cancer patients undergoing adjuvant gemcitabine chemotherapy [21, 24]. In the present study, median RFS was 34.95 months for DCK-positive patients, compared with 11.41 months for DCK-negative patients. These findings suggest the possibility of individualized therapy for BTC based on pharmacogenomic markers.

Several genetic and epigenetic alterations, including gene mutations, amplifications, polymorphic status or altered gene/protein expression and activity, have been associated with gemcitabine response and toxicities [20, 25]. In our previous retrospective study of patients with advanced BTC who were treated with gemcitabine plus cisplatin, the variant rs1048977 allele in the CDA gene was associated with tumor response in a dominant model [14]. In the present study, however, none of the tested SNPs was significantly associated with RFS or with hematologic or non-hematologic toxicities. Prospective studies with larger sample sizes are needed to evaluate the role of genetic markers in patients with BTC [26].

This study had several other limitations. The first was the lack of a concurrent control arm. However, concerns regarding sample size, the availability of patients with this rare disease entity, and the ability to complete the trial in a timely fashion indicated the need for a single-arm study. Second, the pattern of failure after resection, as well as tumor biology, may differ according to tumor origin, with local failure regarded as a prominent feature of extrahepatic cholangiocarcinoma but not of gallbladder cancer or intrahepatic cholangiocarcinoma [27]. Although there was no difference in gemcitabine efficacy among disease subsites, the results should be interpreted with caution. Positive dCK expression were observed in 8/31 (26%) in extrahepatic cholangiocarcinoma, 8/22 (36%) in GB cancer, and 5/13 (38%) in intrahepatic cholangiocarcinoma. Due to the small number of event, further reduction in sample size may not have enough power to any potential difference. Furthermore, our trial may not have had sufficient power to show associations between genetic polymorphisms and gemcitabine efficacy or toxicity. Although the primary endpoint of this study was not met, intratumoral DCK expression was significantly associated with RFS of patients with resectable BTC treated with postoperative gemcitabine chemotherapy. Future randomized controlled trials are warranted testing therapeutic strategies for adjuvant gemcitabine in patients resected for BTC.

## MATERIALS AND METHODS

### Study design and patients

This prospective, phase II clinical trial was open for accrual from July 2009 through July 2014 after approval by the Institutional Review Board of the National Cancer Center, Korea (IRB No. NCCCTS-09-411). The trial included patients who had undergone surgical resection for BTC (but not T1 gallbladder cancer or ampullary cancer) with curative intent within 4 months of staging. Patients had to have recovered from surgery and have Eastern Cooperative Oncology Group (ECOG) performance status (PS) of 0 or 1. All patients underwent computed tomography (CT) or magnetic resonance imaging (MRI) of the chest, abdomen, and pelvis within 4 weeks before registration to rule out distant metastasis. Patients were excluded if they had received prior anticancer therapy for the current malignancy or upper abdominal radiotherapy at any time. Patients had to have an absolute neutrophil count ≥ 1,500/mm3, a platelet count ≥ 100,000/mm3, serum creatinine ≤ 1.5 X the upper limit of normal, total bilirubin ≤ 1.5 mg/dl, and either AST or ALT 2× the upper limit of normal.

Within 8 weeks after gross complete resection of BTC, the patients were started on intravenous gemcitabine (1000 mg/m^2^), administered as a 30-min infusion on days 1, 8, and 15 of every 28 day cycle. Patients were evaluated for disease recurrence by chest X-ray and CT or MRI of the abdomen every 12 weeks. RFS was defined as the time from the first day of study drug administration to tumor recurrence or date of last follow up. Objective recurrence was defined as the appearance of new areas of local and/or distant disease on radiological imaging; as an increase in carbohydrate antigen (CA) 19-9 in the setting of enlarged abdominal, pelvic or retroperitoneal lymph nodes; or any new or enlarged peritoneal, liver or lung mass. Patients with a normal CT scan or MRI and increased CA 19-9 could be assessed by PET scanning to provide evidence for recurrent disease. Although increased serum CA 19-9 alone did not constitute evidence of recurrence, it could prompt re-evaluation by acceptable imaging modalities. Imaging tests were also repeated when clinically indicated (e.g., to confirm disease recurrence).

**Figure 3:**
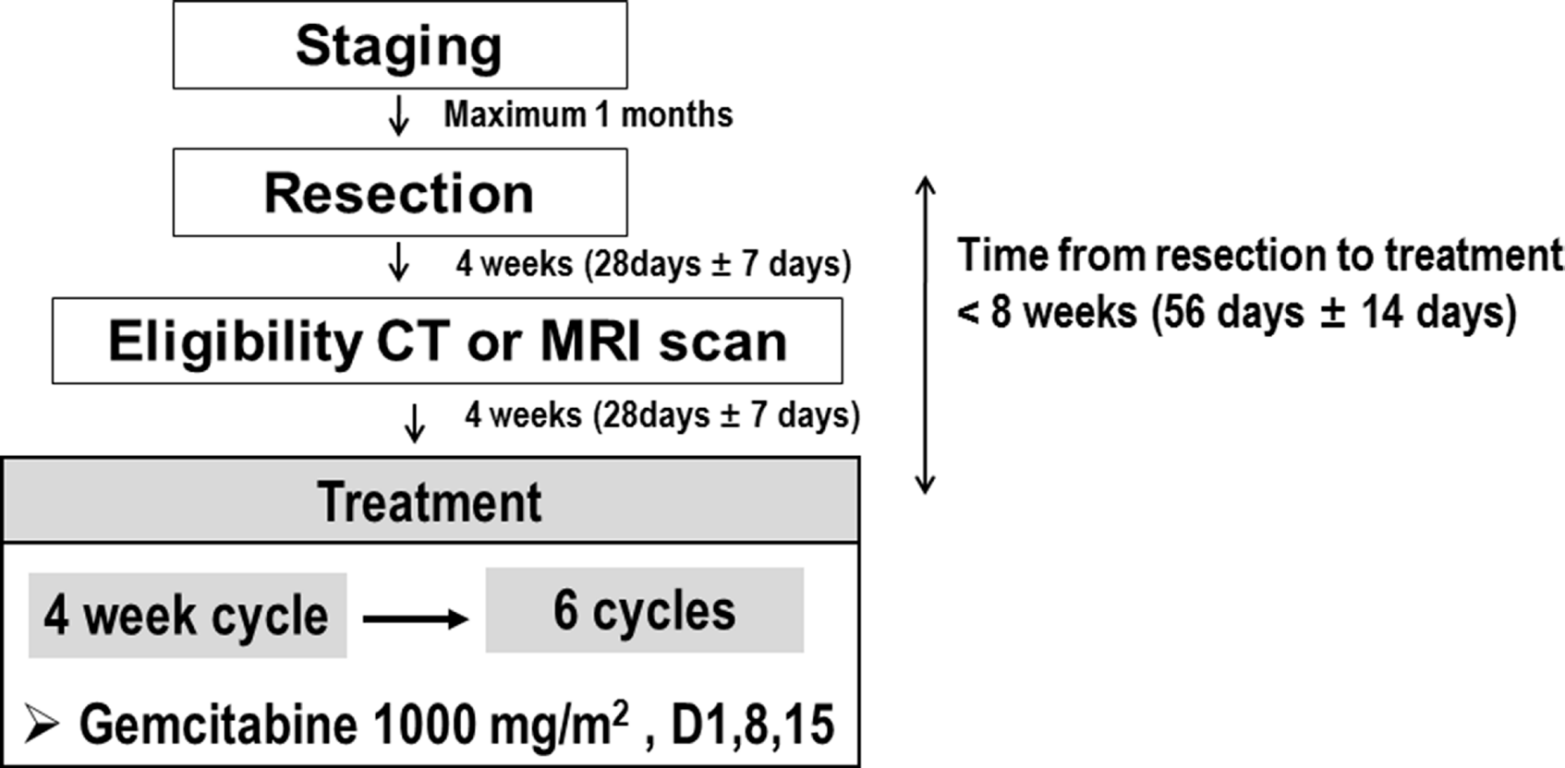
Study scheme and treatment schedule

The study scheme and gemcitabine treatment schedule are shown in Figure 3. Patients were discontinued if there was evidence of disease recurrence, unacceptable toxicity, or a need for any treatment not allowed by the protocol. Patients who elected to discontinue treatment for any reason and those who did not comply with study procedures were also withdrawn. Doses were reduced and/or delayed if patients experienced severe hematologic and/or non-hematologic toxicities while on study treatment. Doses were adjusted according to the system showing the greatest degree of toxicity. All patients who received at least one dose of study drug were included in the safety population. Safety analyses were based on laboratory test abnormalities and on clinical adverse events. Toxicities were graded using the CTCAE 4.1.

### Preparation and immunostaining of surgical specimens

All surgical specimens were independently examined by two pathologists. Tumors were classified as well-differentiated, moderately differentiated, or poorly differentiated adenocarcinomas, based on predominant pathological grading. Pancreatic invasion, duodenal invasion, hepatic invasion, vascular invasion and lymph node status were assessed for all surgical specimens. Surgical margins were graded R1 if residual microscopic tumor cells were identified in the proximal or distal bile duct transaction line, the hepatic transaction line, or the dissected periductal soft tissue margins. The final stage of BTC was graded on the basis of the UICC tumor-node-metastasis classification, 7th edition. Hematoxylin and eosin-stained slides containing specimens from each BTC sample were reviewed, and a representative tumor region and the corresponding formalin-fixed paraffin-embedded tissue block were selected for immunohistochemistry.

**Figure 4:**
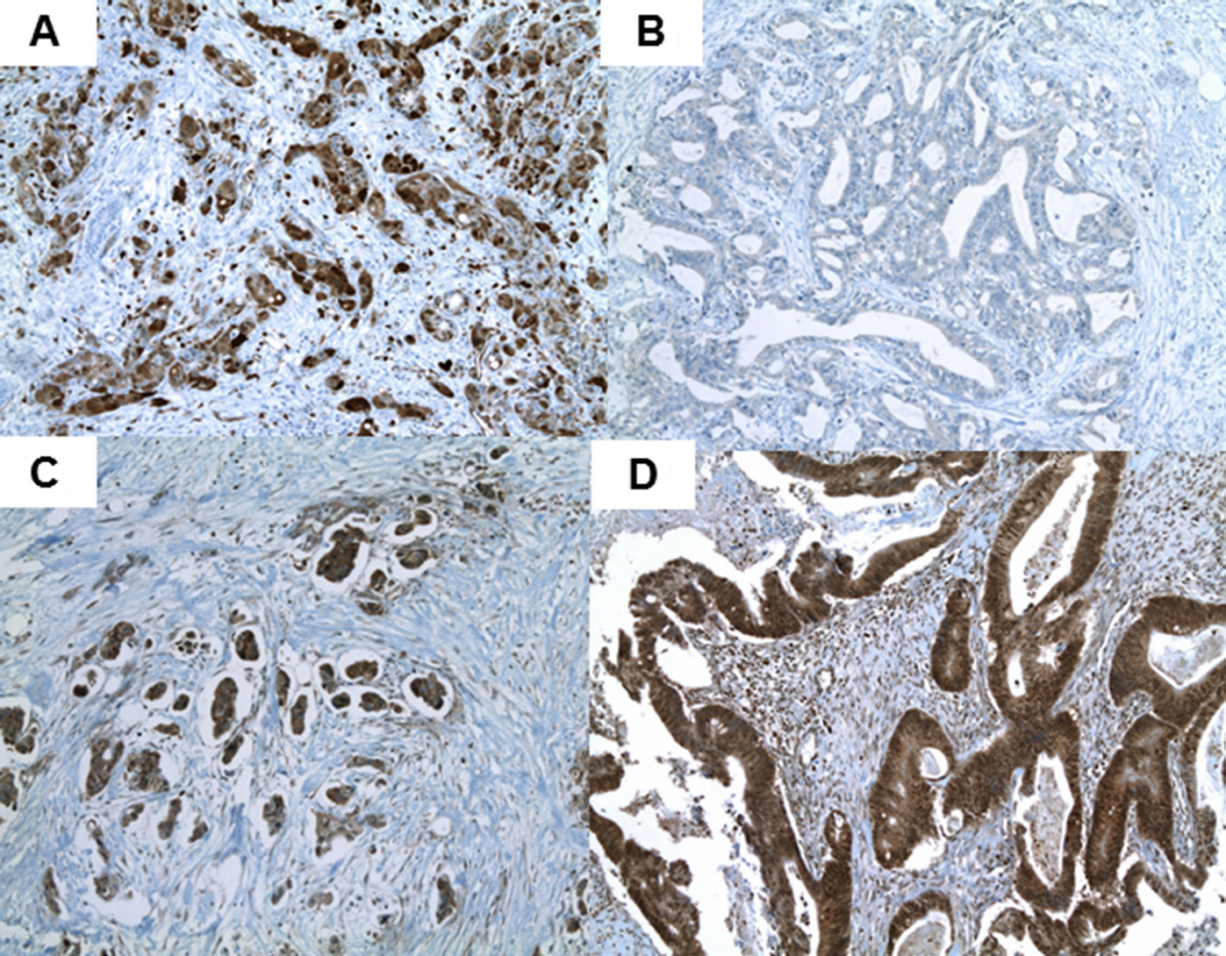
Typical positive immunohistochemical labeling profiles of CDA (**A**), dCK (**B**), hENT1 (**C**), and RRM1 (**D**) in biliary tract cancer.

One to three paraffin-embedded blocks (median, two blocks) of each resected specimen were used for immunohistochemistry. Three serial 3-μm sections were cut and prepared from each block: one for hematoxylin-eosin staining; one for immunohistochemical staining with the indicated primary antibodies; and one being a negative control. Two pathologists (W.S.P. and E.K.H; blinded to clinical characteristics and outcomes) assessed the staining patterns and immunoreactivities of proteins associated with gemcitabine transport and metabolism (hENT1, dCK, RRM1 and CDA). When the observers differed in their findings, they jointly re-investigated the slides and arrived at a consensus. Briefly, formalin-fixed, paraffin-embedded BTC sections were deparaffinized with xylene and rehydrated with a graded series of aqueous ethanol. For antigen retrieval, slides were heated at 98°C in 10 mM/L citrate buffer (pH 6.0) for 15 minutes using the Microwave Processing Labstation for Histology (Micromed T/T MEGA, Sorisole, Italy). Endogenous peroxidase activity was blocked with 3% hydrogen peroxide, and sections were incubated at 42°C for 32 minutes with rabbit polyclonal antibody to human CDA (ProSci, Inc., Poway, CA, USA), human dCK (LifeSpan Bioscience, Inc., Seattle, WA, USA), human hENT1 (ProteinTech Group, Inc., Chicago, IL, USA), or human RRM1 (ProteinTech Group, Inc.). The slides were rinsed twice with Tris buffer, incubated with biotin (iVIEW DAB detection kit, Ventana, Tucson, AZ, USA) for 10 minutes, rinsed again, and incubated with streptavidin for 8 minutes. After a final rinse with Tris buffer, the chromogen (dimethylaminoazobenzene, Ventana) was applied for 8 minutes, followed by copper solution for 4 minutes. Slides were counterstained with commercially prepared hematoxylin for 4 minutes. Following post-counterstaining with bluing solution, the slides were dehydrated and coverslipped with Permount (Fisher). BenchMark XT (Ventana) was used for all staining. As a negative control, irrelevant primary antibodies were used. Immunohistochemical labeling of hENT1, dCK, RRM1 and CDA was scored as positive, indicating the presence of an intact gene, or negative, indicating a deletion or inactivating mutation of that gene (Figure 4, Supplementary Figure 2). Cellular staining was localized to the membrane for hENT1, the cytoplasm for dCK and CDA, and the nucleus for RRM1. Levels of expression of hENT1 and dCK were defined with reference to lymphocytes, the internal positive control, as described [28, 29].

### SNP selection and genotyping

Eight SNPs in the genes encoding CDA, DCK, hENT1, human concentrative nucleoside transporter 3 (hCNT3) and RRM1 were selected (for details, see Supplementary Table 2). Genomic DNA was extracted from whole blood samples using QIAamp DNA Blood Mini kits (Qiagen, Valencia, CA, USA), according to the manufacturer's instructions. The polymorphisms were genotyped using the TaqMan 5′ nuclease assay for allelic discrimination, commercially available TaqMan probes, and a 384-well ABI 7900HT Sequence Detection System (all from Applied Biosystems, Foster City, CA, USA). To confirm genotyping results, the genotyping reactions for 10% of the samples were retested. Genotyping reactions were performed using 10 ng genomic DNA, 2x TaqMan Universal PCR Master Mix (Applied Biosystems), and the appropriate probes and primers in a 384-well plate. The amplification protocol consisted of an initial denaturation at 95°C for 10 min, followed by 40 cycles of denaturation at 95°C for 15 s and annealing and extension at 60°C for 1 min. The genotyping results were analyzed by allelic discrimination plots using SDS 2.1 software (version 5.0, Applied Biosystems).

### Statistical analysis

The primary endpoint of the study was the 2 year RFS rate. The expected rate following curative resection was approximately 45%, except for patients with stage I gallbladder cancer [14]. Following gemcitabine treatment, the expected 2-year RFS rate was about 60%. Sample size was calculated for a single arm study, with 65 patients required to detect this difference with 80% power and a two-sided type I error rate of 5%. Considering a 10% drop-out rate, the goal was to recruit 72 patients over 24 months.

The 2-year RFS rate and median time to recurrence were estimated using the Kaplan-Meier method. The associations between RFS and clinicopathologic characteristics were analyzed using univariable or multivariable Cox proportional-hazards regression models. Due to the small number of events (and small sample size), variables assessed in multivariable analysis were pre-selected based on the result of univariable analysis (*P* < 0.2). These variables were entered into the multivariable model, with the final model selected using a backward variable selection method with an elimination criterion of *p*-value > 0.05. The effects of intratumoral protein expression on RFS were evaluated by adding the expression of each protein expression as an explanatory variable to the multivariable Cox model. The association of each SNP with RFS and toxicity (grade ≥ 3) was analyzed using a Cox proportional hazard model for RFS and logistic regression for toxicity. A dominant genetic model for each SNP was considered. The hazard ratios (HRs) for the Cox proportional hazard model and the odds ratios (ORs) for the logistic model were reported together with their associated 95% confidence intervals (CIs). All analyses were performed using SAS statistical software (version 9.3) and R (version 3.3.1) and reported *p*-values are two-sided.

## SUPPLEMENTARY MATERIALS FIGURES AND TABLES


