# Supplementary material for: DCK expression, a potential predictive biomarker in the adjuvant gemcitabine chemotherapy for biliary tract cancer after surgical resection: results from a phase II study
Source: Oncotarget. 2017 Jul 6;8(46):81394–404. doi: 10.18632/oncotarget.19037 (PMC5655294; doi:10.18632/oncotarget.19037)
Supplement: Supplementary file 1 [file oncotarget-08-81394-s001.pdf]

DCK expression, a potential predictive biomarker in the adjuvant gemcitabine chemotherapy for biliary tract cancer after surgical resection: results from a phase II study

Supplementary Materials

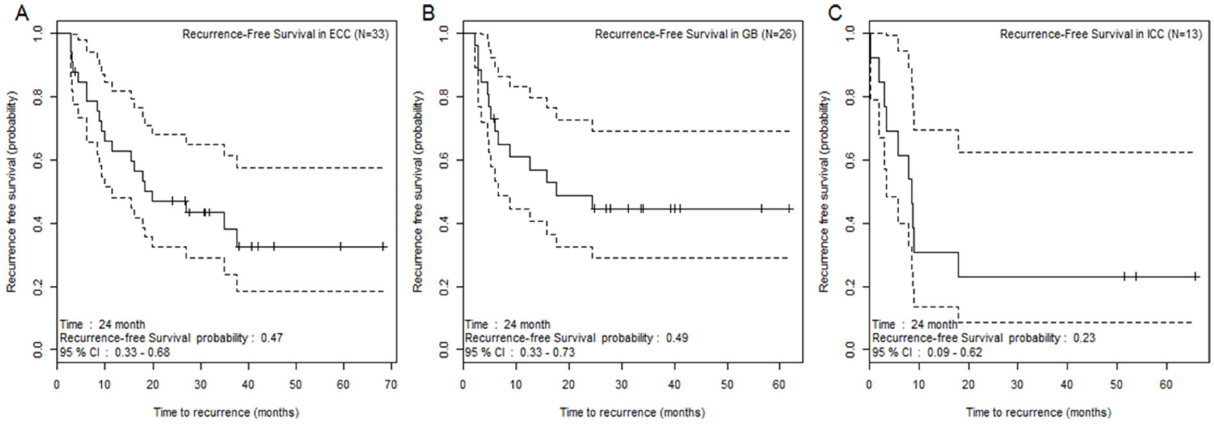

Supplementary Figure 1: Estimates of 2-year recurrence-free survival in extrahepatic cholangiocarcinoma (**A**), gallbladder cancer (**B**) and intrahepatic cholangiocarcinoma (**C**).

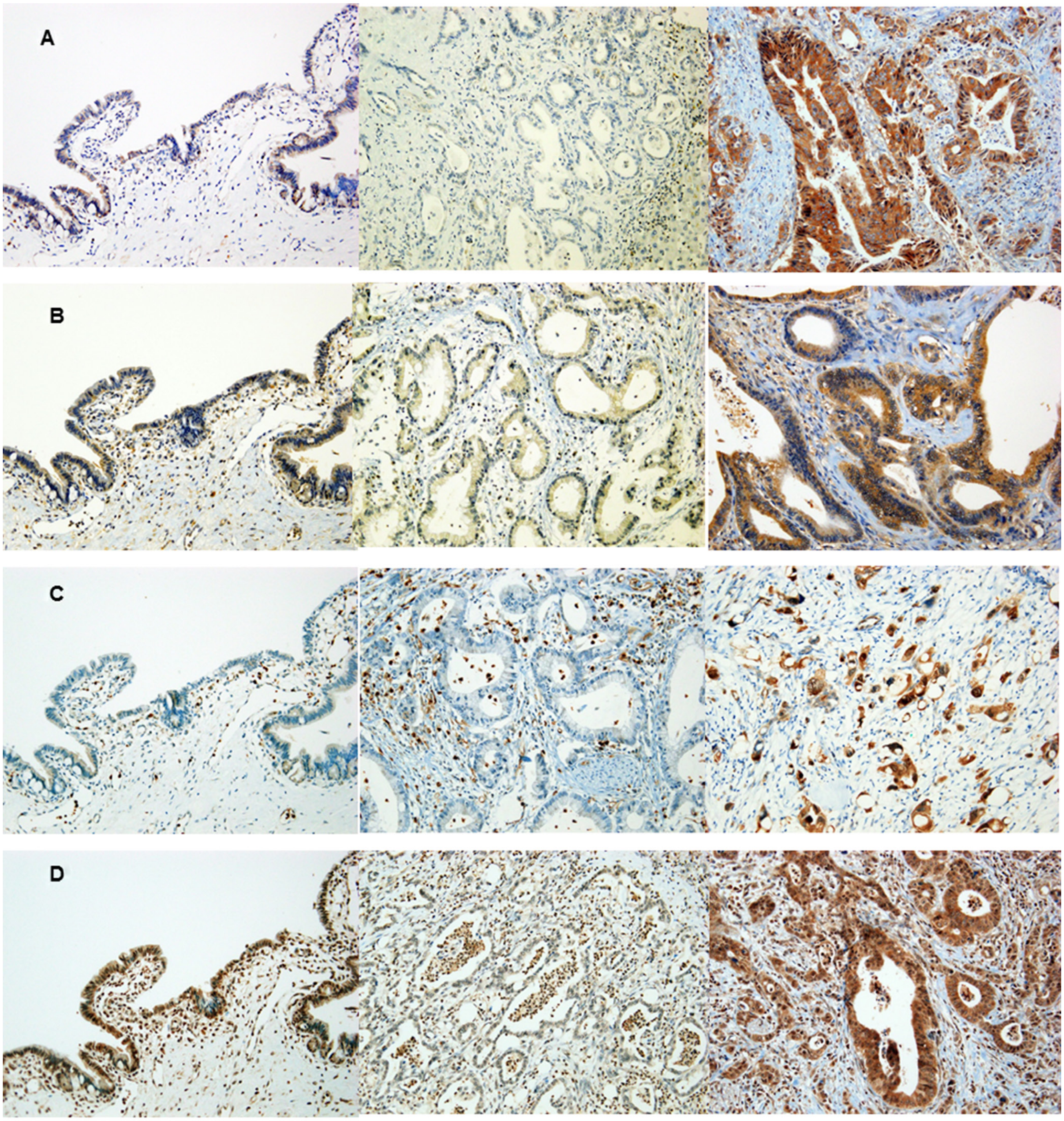

Supplementary Figure 2: Immunohistochemical staining of normal bile duct mucosa, biomarker-negative and biomarker-positive cholangiocarcinoma, resepectively. (**A**) CDA, (**B**) dCK, (**C**) hENT1 and (**D**) RRM1.

Supplementary Table 1: Distribution of Adverse Event of patients with and without recurrence

	N (%)	Non-Recurrence	Recurrence	
**Characteristic**		*N* (%)	*N* (%)	***P***
No. of Patients	72	44	28	
Nausea				0.6422‡
0: grade < 3	67 (93.06)	27 (96.43)	40 (90.91)	
1: grade ≥ 3	5 (6.94)	1 (3.57)	4 (9.09)	
Vomiting				0.5180‡
0: grade < 3	70 (97.22)	28 (100)	42 (95.45)	
1: grade ≥ 3	2 (2.78)	0(0)	2 (4.55)	
Anorexia				1.0000 ‡
0: grade < 3	70 (97.22)	27 (96.43)	43 (97.73)	
1: grade ≥ 3	2 (2.78)	1 (3.57)	1 (2.27)	
Diarrhea				1.0000 ‡
0: grade < 3	70 (97.22)	27 (96.43)	43 (97.73)	
1: grade ≥ 3	2 (2.78)	1 (3.57)	1 (2.27)	
Febrile_neutropenia				1.0000 ‡
0: grade < 3	71 (98.61)	28 (100)	43 (97.73)	
1: grade ≥ 3	1 (1.39)	0(0)	1 (2.27)	
Abdominal_pain				0.5180 ‡
0: grade < 3	70 (97.22)	28 (100)	42 (95.45)	
1: grade ≥ 3	2 (2.78)	0(0)	2 (4.55)	
Asthenia				1.0000 ‡
0: grade < 3	71 (98.61)	28 (100)	43 (97.73)	
1: grade ≥ 3	1 (1.39)	0(0)	1 (2.27)	
ALT				1.0000 ‡
0: grade < 3	71 (98.61)	28 (100)	43 (97.73)	
1: grade ≥ 3	1 (1.39)	0(0)	1 (2.27)	
AST				1.0000 ‡
0: grade < 3	71 (98.61)	28 (100)	43 (97.73)	
1: grade ≥ 3	1 (1.39)	0(0)	1 (2.27)	
T_bilirubin				0.3705‡
0: grade < 3	67 (93.06)	25 (89.29)	42 (95.45)	
1: grade ≥ 3	5 (6.94)	3 (10.71)	2 (4.55)	
Dizziness				1.0000 ‡
0: grade < 3	71 (98.61)	28 (100)	43 (97.73)	
1: grade ≥ 3	1 (1.39)	0(0)	1 (2.27)	
Pneumonitis				1.0000 ‡
0: grade < 3	70 (97.22)	27 (96.43)	43 (97.73)	
1: grade ≥ 3	2 (2.78)	1 (3.57)	1 (2.27)	
Anemia				1.0000 ‡
0: grade < 3	68 (94.44)	27 (96.43)	41 (93.18)	
1: grade ≥ 3	4 (5.56)	1 (3.57)	3 (6.82)	
Platele.decreased				0.5559‡
0: grade < 3	69 (95.83)	26 (92.86)	43 (97.73)	
1: grade ≥ 3	3 (4.17)	2 (7.14)	1 (2.27)	
Neutrophil.count.decreased				0.4492 †
0: grade < 3	32 (44.44)	14 (50)	18 (40.91)	
1: grade ≥ 3	40 (55.56)	14 (50)	26 (59.09)	

†Pearson's chi-square test.
‡Fisher's Exact test.

Supplementary Table 2: Association between SNPs and hazard rate of recurrence

Gene	SNP	Variation		Number			Dominant	
				V/V	V/W	W/W	HR (95% CI)	*p* value
CDA	rs602950	–92	A > G	1	15	34	1.98 (0.91–4.29)	0.0849
	rs1048977	435	C > T	3	18	29	1.64 (0.77–3.49)	0.2029
DCK	rs67437265	364	C > T	0	8	42	0.27 (0.06–1.15)	0.0768
hENT1	rs747199	–706	G > C	2	14	34	0.97 (0.43–2.16)	0.9312
hCNT3	rs56350726	1538	A > T	0	5	45	0.36 (0.05–2.66)	0.3177
RRM1	rs11030918	–756	T > C	1	19	27	0.44 (0.18–1.06)	0.0661
	rs12806698	–269	C > A	2	18	29	0.51 (0.22–1.17)	0.1124
	rs183484	850	C > A	13	20	16	1.73 (0.69–4.31)	0.2413

None of the 8 SNPs had significant association with RFS.

Supplementary Table 3: Association between SNPs and Grade 3 or higher hematologic or non-hematologic toxicities

Gene	SNP	Variation		Number			Hematology		Non-hematology	
				V/V	V/W	W/W	OR (95% CI)	*p* value	OR (95% CI)	*p* value
CDA	rs602950	–92	A > G	1	15	34	1.17 (0.34–3.96)	0.8046	0.16 (0.02–1.38)	0.0955
	rs1048977	435	C > T	3	18	29	1.15 (0.36–3.62)	0.8151	0.23 (0.05–1.23)	0.0855
DCK	rs67437265	364	C > T	0	8	42	0.62 (0.14–2.81)	0.5311	1.22 (0.21–7.12)	0.8233
hENT1	rs747199	–706	G > C	2	14	34	0.80 (0.24–2.66)	0.7106	1.29 (0.32–5.24)	0.7257
hCNT3	rs56350726	1538	A > T	0	5	45	1.00 (0.15–6.59)	1.0000	2.67 (0.39–18.42)	0.3199
RRM1	rs11030918	–756	T > C	1	19	27	0.72 (0.22–2.33)	0.5828	0.32 (0.06-1.73)	0.1847
	rs12806698	–269	C > A	2	18	29	0.53 (0.16–1.69)	0.2798	0.46 (0.11–2.02)	0.3059
	rs183484	850	C > A	13	20	16	1.20 (0.36–4.01)	0.7712	0.81 (0.20–3.30)	0.7659

None of the 8 SNPs had significant association with toxicity of hematology & non-hematology variables.

Supplementary Table 4: SNPs evaluated in this study

Gene	Chromosome	SNP	Reference SNP ID number
CDA	1p36.12		
		-92 A > G	rs602950
		435 C > T	rs1048977
DCK	4q13.3	364 C > T	rs67437265
hENT1	6p21.1	–706 G > C	rs747199
hCNT3	9q21.32	1538 A > T	rs56350726
RRM1	11p15.4	–319-437 T > C	rs11030918
		–269 C > A	rs12806698
		850 C > A	rs183484
